# Overexpression of the Large-Conductance Mechanosensitive Channel Gene in *Oenococcus oeni* Enhances Its Ethanol Stress Tolerance

**DOI:** 10.3390/microorganisms14050973

**Published:** 2026-04-26

**Authors:** Longxiang Liu, Yang Zhao, Lemeng Zhang, Yujuan Zheng, Shuai Peng, Hongyu Zhao, Xinyu Zhao, Yumiao Zhang, Jingjing Fang, Weiyu Song

**Affiliations:** 1 Binzhou Key Laboratory of Edible Fungi Breeding and High Value Utilization (Preparation), College of Biological and Pharmaceutical Engineering, Shandong University of Aeronautics, Binzhou 256600, China; longxiang8802@163.com (L.L.); zhy3467@foxmail.com (Y.Z.); 18963087102@163.com (L.Z.); zhengyujuan9003@163.com (Y.Z.); 16688089770@163.com (X.Z.); zhangyumiao_163@163.com (Y.Z.); 2 Shandong Key Laboratory of Eco-Environmental Science for the Yellow River Delta, Binzhou 256600, China; 3 Gansu Province Wine Industry Technology Research and Development Center, College of Food Science and Engineering, Gansu Agricultural University, Lanzhou 730070, China; pengshuai1987@163.com; 4 College of Enology, Northwest A&F University, Xinong Road 22, Yangling 712100, China; hongyuzhao@nwafu.edu.cn

**Keywords:** *Oenococcus oeni*, ethanol stress, overexpression, proteome

## Abstract

*Oenococcus oeni* (*O. oeni*) can initiate and complete the malolactic fermentation (MLF) process, which significantly improves wine quality. However, stress factors commonly encountered in wine, such as acid stress and ethanol stress, can hinder this process. Overexpression of certain key functional genes using genetic recombination technology can enhance the stress tolerance of *O. oeni*. In this study, the large-conductance mechanosensitive channel (*mscl*) gene was overexpressed in *O. oeni* SD-2a using genetic recombination technology. The results showed that overexpression of this gene enhanced the growth rate of *O. oeni* under 10% ethanol stress conditions. Physiological index measurements indicated that overexpression of this gene enhanced the control of cell membrane permeability in the recombinant strain at different time points under ethanol stress and altered cell membrane fluidity at these time points. Proteomic analysis after 12 h of treatment under 10% ethanol stress revealed that *mscl* overexpression significantly altered the protein expression pattern of *O. oeni*. The most significantly affected proteins included some cell membrane transporters (for sugars, lipids, amino acids, and nucleotides) and proteins involved in cell wall synthesis. These results suggest that *mscl* overexpression enhances the ethanol stress tolerance of *O. oeni* by altering its cell membrane properties and affecting the expression levels of proteins related to cell membrane transport and cell wall synthesis. This study provides a theoretical reference for obtaining *O. oeni* recombinant strains with enhanced stress tolerance through genetic recombination technology.

## 1. Introduction

Various fermented alcoholic beverages, including wine, are favored by young consumers and have promising market growth prospects [1]. Taking wine production as an example, the process involves alcoholic fermentation dominated by *Saccharomyces cerevisiae* and malolactic fermentation (MLF) dominated by *Oenococcus oeni* (*O. oeni*) [2,3]. The latter significantly improves the taste of wine by reducing acidity and astringency while increasing smoothness [4]. Furthermore, because MLF catalyzes the conversion of the dicarboxylic acid malate into the monocarboxylic acid lactate, it enhances the microbial stability of the wine and reduces the risk of spoilage [5]. Additionally, *O. oeni* can produce aromatic substances such as volatile esters and diacetyl through its own metabolic processes, giving the wine a rich flavor [6]. Therefore, *O. oeni* and the MLF it drives are considered essential for improving wine quality.

During fermentation, *O. oeni* encounters stress factors such as low pH, high ethanol concentration, high SO_2_ concentration, and suboptimal growth temperatures [7]. These stress factors can significantly inhibit the growth and activity of *O. oeni* and can substantially hinder or delay the onset of MLF [7]. Therefore, identifying robust *O. oeni* strains for application in wine production is of great importance. In addition to screening superior strains from natural environments, strain improvement technologies can also be used to obtain strains with desirable traits. These include adaptive laboratory evolution to select mutants with enhanced stress tolerance, mutagenesis techniques to obtain mutants with increased stress tolerance, and genetic modification techniques to introduce exogenous beneficial genes to enhance the stress tolerance of recombinant strains [8]. Betteridge et al. (2018) were the first to obtain an *O. oeni* strain with high ethanol tolerance using directed evolution [9]. This strain completed MLF in a high-ethanol-concentration medium in a shorter time than the original strain, and the expression level of the *hsp18* gene was increased by 20%. Li et al. (2015) used ultraviolet mutagenesis to screen for a strain with high MLF activity, which showed a 38.81% increase in fermentation capacity, and the characteristics of the wine produced by the mutant strain were improved [10]. Furthermore, heterologous expression of *O. oeni* genes, such as *argG*, *trxA*, and the two-component signal transduction response regulator in *Lactobacillus plantarum* significantly enhanced the stress tolerance of the recombinant strains [11,12,13]. These techniques have laid a foundation for obtaining superior strains for industrial production.

Our research group previously obtained an *O. oeni* mutant with enhanced acid stress tolerance using directed evolution. Through genome sequencing and comparative genomics, we identified the large-conductance mechanosensitive channel gene as potentially influencing acid stress tolerance in *O. oeni*. Studies have shown that Mscl is widely present in microorganisms and can sense pressure or osmotic stress [14]. This protein was first discovered in *Escherichia coli*, and its crystal structure was resolved in *Mycobacterium tuberculosis* [15]. The protein exists as a homopentamer, with each pentamer subunit consisting of two transmembrane helices (TM1 and TM2) and a third cytoplasmic helix. When the membrane is stretched, Mscl responds to increased membrane tension and opens a non-selective pore approximately 30 Å wide [15,16]. This process acts as a pressure relief valve, protecting the cell from lysis during acute osmotic downshock [16]. Point mutation experiments have shown that mutant proteins with substitutions at key amino acid residues exhibit phenotypes such as slow growth and leakage of cytoplasmic solutes [17]. Changes in pH may lead to the ionization of charged residues in Mscl or lipid head groups, causing conformational changes in the Mscl protein and hydrophobic mismatch between the protein and lipids [18]. The specific physiological functions of Mscl in different microorganisms require further investigation.

In this study, we constructed an *O. oeni mscl* overexpression vector, pIB184-*Oenomscl*, using genetic recombination technology. This vector was introduced into *O. oeni* SD-2a via electroporation to obtain the *Oenomscl* overexpression strain. Through physiological index measurements and proteomic data analysis, we investigated the specific effects of *Oenomscl* overexpression on *O. oeni* under stress conditions, laying a foundation for clarifying its specific function in *O. oeni*.

## 2. Materials and Methods

### 2.1. Strains and Culture Conditions

*Oenococcus oeni* SD-2a was provided by the College of Enology, Northwest A&F University. The control strain (control, transformed with the empty pIB184 vector) and the recombinant strain (transformed with pIB184-*Oenomscl*) were activated and cultured. They were inoculated into FMATB medium (glucose 5 g/L, D, L-malate 5 g/L, yeast extract 5 g/L, peptone 10 g/L, MgSO_4_·7H_2_O 0.2 g/L, MnSO_4_·4H_2_O 0.05 g/L, Cysteine/HCl 0.5 g/L, and tomato juice 250 mL) [19] containing 20 μg/mL erythromycin, either under acid stress conditions (pH 3.2, pH 3.4, pH 3.6, adjusted by 1 M HCl) without ethanol or ethanol stress conditions (10%, 12%, 14% *v*/*v* ethanol, at pH 4.8), and incubated statically at 30 °C.

### 2.2. Construction of Recombinant Plasmid and Recombinant Strain

A frozen glycerol stock of *O. oeni* SD-2a was streaked onto an FT80 solid plate. A single colony was picked and cultured until the culture was turbid. Genomic DNA was extracted. Primers for amplifying the *Oenomscl* fragment were designed: Oenomscl-up: CAATGATGTTGGATCCATGTTAAATGAATTTAAGCAGTTTATTATGCGTGGA, Oenomscl-down: TCGAGCTCTAGAATTCTTAGGCTTTGTTCGTGGATTGATTGGA. The *Oenomscl* fragment was amplified by PCR, purified, and recovered. The pIB184 vector and the *Oenomscl* fragment were double-digested with *Eco*RI and *Bam*HI. The resulting linear vector and fragment were ligated to obtain the recombinant plasmid. The recombinant plasmid was transformed into *Escherichia coli* Top10 competent cells. Positive clones were identified using colony PCR. The recombinant plasmid from a positive clone with confirmed sequencing was extracted and transformed into *O. oeni* SD-2a cells by electroporation. Successfully transformed recombinant strains were identified by colony PCR for subsequent experiments. The empty pIB184 vector was extracted and transformed into *O. oeni* SD-2a cells by electroporation to obtain the control strain.

### 2.3. Growth Curve Measurement

The control strain and *Oenomscl* overexpression strain, activated and grown to an OD_600_ of 0.6, were inoculated at 1% (*v*/*v*) into FMATB medium under the corresponding conditions. Samples were taken every 12 h, and the absorbance at 600 nm was measured using a UV-Vis spectrophotometer (UV-5500, Shanghai Yuanjie Instrument Co., Ltd., Shanghai, China) to plot the growth curves.

### 2.4. Cell Membrane Fluidity Measurement

The determination method was similar to that in reference [13] with slight modifications. Briefly, the control strain and *Oenomscl* overexpression strain were grown under 10% ethanol stress until the designated time points. An appropriate volume of culture was taken, and the cell concentration was adjusted to an OD_600_ of 0.6. Formaldehyde was added to a final concentration of 0.25% and cells were incubated at 30 °C for 30 min for fixation. After centrifugation, the cell pellet was washed twice with 0.2 M phosphate buffer (pH 7.4) containing 0.25% formaldehyde. To measure lateral diffusion, cells were labeled with 1 μL pyrene at 30 °C for 40 min. Fluorescence intensity was measured using a fluorescence spectrophotometer (F4700, Hitachi, Tokyo, Japan) with excitation at 335 nm and emission at 373 nm (monomer) and 470 nm (excimer), and a slit width of 5 nm. Membrane fluidity was calculated based on probe concentration and the ratio of fluorescence intensity at 470 nm to that at 373 nm.

### 2.5. Cell Membrane Permeability Measurement

The determination method was similar to that in reference [13] with slight modifications. The control strain and *Oenomscl* overexpression strain were grown under 10% ethanol stress until the designated time points. An appropriate volume of culture was centrifuged to obtain cell pellets. Cells were resuspended in 10 mmol/L phosphate-buffered saline (pH 7.4) and the OD600 was adjusted to 1.0. Then, 10 μL of 5 mmol/L p-nitrophenyl-α-D-galactopyranoside (pNPG) was added and the whole mixture was incubated at 30 °C. After 2 h, the absorbance was measured at 420 nm using a UV-visible spectrophotometer (UV-5500, Shanghai, China).

### 2.6. Cell Membrane Integrity Measurement

The determination method was similar to that in reference [13] with slight modifications. The control strain and *Oenomscl* overexpression strain were grown under 10% ethanol stress until the designated time points. An appropriate volume of culture was centrifuged to obtain cell pellets. Cells were washed twice with phosphate-buffered saline (pH 7.4) and resuspended to an OD_600_ of 0.6. Propidium iodide (PI) was added to a final concentration of 50 μg/mL, and the mixture was incubated for 30 min at 30 °C in the dark. Fluorescence was measured using a multimode microplate reader (Tecan, Männedorf, Switzerland) with excitation at 488 nm and emission at 630 nm.

### 2.7. Real-Time Quantitative PCR

The determination method was similar to that in reference [12]. The Bacteria RNA Extraction Kit (Vazyme Biotech, Nanjing, China) and HiScript III 1st Strand cDNA Synthesis Kit (Vazyme Biotech) were used to extract RNA and reverse-transcribe it into complementary DNA. Real-time quantitative PCR (RT-qPCR) was performed on a Real-Time PCR Detection System (Thermo Fisher Scientific, Cleveland, OH, USA) using AceQ qPCR SYBR Green Master Mix (Vazyme Biotech). Relative expression levels were calculated by the 2^−ΔΔCt^ method using 16S ribosomal RNA as the reference gene and *Oenomscl* overexpression strain as the control strain.

### 2.8. Proteomic Data Determination and Analysis

The control strain and *Oenomscl* overexpression strain were grown under 10% ethanol stress for 12 h. Cell pellets were obtained by centrifugation at 13,700× *g* for 5 min and stored at −80 °C. The samples were sent to Shanghai Majorbio Bio-pharm Technology Co., Ltd. (Shanghai, China) for proteomic data determination. All samples were transferred to MP tubes while frozen. An appropriate amount of protein lysis buffer (8 M urea + 1% SDS, containing protease inhibitors) was added. The samples were agitated three times for 180 s each using a high-throughput tissue grinder, followed by non-contact low-temperature sonication for 30 min. After centrifugation at 14,000× *g* for 15 min at 8 °C, the supernatant was collected. Protein concentration was determined using the BCA method. After protein quantification, SDS-PAGE was performed. 100 μg of protein sample was taken, lysis buffer was added, and 100 mM triethylammonium bicarbonate buffer (TEAB) was added to a final concentration. A total of 10 mM tris(2-carboxyethyl)phosphine (TCEP) was added, and the mixture was incubated at 37 °C for 60 min. A total of 40 mM iodoacetamide was added and incubated at room temperature in the dark for 40 min. Pre-chilled acetone was added to each tube (acetone:sample *v*:*v* = 6:1), and proteins were precipitated at −20 °C for 4 h. After centrifugation at 10,000× *g* for 20 min, the pellet was collected and fully dissolved in 100 µL of 100 mM TEAB. Trypsin was added at a 1:50 (enzyme:protein) ratio for overnight digestion at 37 °C. After digestion, the peptides were dried under vacuum. The dried peptides were reconstituted in 0.1% trifluoroacetic acid (TFA), desalted using HLB cartridges, and dried again in a vacuum concentrator. Peptide quantification was performed using UV spectrophotometry (NANO DROP ONE, Thermo Scientific). Equal amounts of peptides were dissolved in mass spectrometry loading buffer for DIA analysis. Peptide separation was performed using a Vanquish Neo chromatograph (Thermo) with a uPAC High Throughput column (75 μm × 5.5 cm, Thermo, USA). Mobile phase A was water (2% acetonitrile + 0.1% formic acid), and mobile phase B was water (80% acetonitrile + 0.1% formic acid). The chromatography runtime was 8 min. The gradient conditions started at 4% B, then a linear gradient to 8% B at 0.1 min, then a linear gradient to 12.5% B at 1 min at a flow rate of 2.5 μL/min, then a linear gradient to 12.6% B at 1.1 min, then a linear gradient to 22.5% B at 3.1 min, then a linear gradient to 45% B at 4.6 min, then a linear gradient to 99% B at 5 min, and 99% B was maintained for the final 3 min at a flow rate of 1.25 μL/min. Data acquisition software was Thermo Xcalibur 4.7 (Thermo, USA). Samples separated by nano-HPLC were analyzed using an Orbitrap Astral mass spectrometer (Thermo) in DIA mode with positive ion detection and an ion source voltage set to 1.5 kV. The mass spectrometry scan range was 100–1700 *m*/*z*. The DIA raw data were imported into the Spectronaut™ 19 software system for database searching. All data were uploaded to the Majorbio Cloud Platform (https://cloud.majorbio.com/, accessed on 19 January 2025) for analysis. The *t*-test function in R language was used to calculate the *p*-value for significance and fold change (FC) between groups. Proteins with *p* < 0.05 and fold change > 1.2 were considered differentially expressed proteins. The KEGG (Kyoto Encyclopedia of Genes and Genomes, http://www.genome.jp/kegg/, accessed on 19 January 2025) pathway database was used to analyze the metabolic pathways involving the differentially expressed proteins.

## 3. Results

### 3.1. Construction of the Oenomscl Overexpression Strain

**Figure 1:**
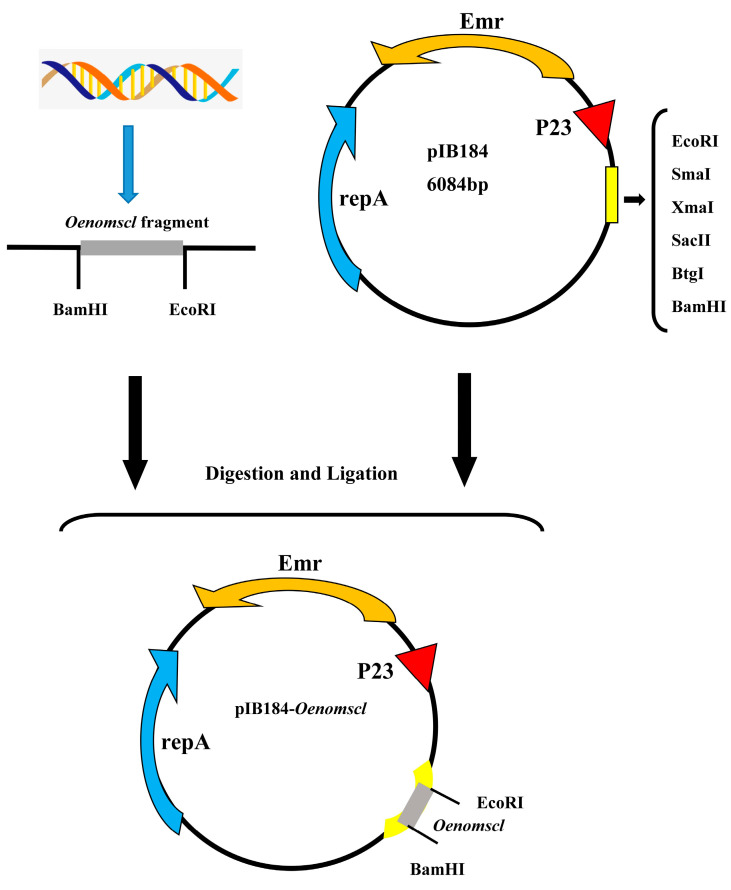
Schematic diagram of the construction process of recombinant plasmid pIB184-*Oenomscl*.

Using the DNA of *O. oeni* SD-2a wild-type strain as a template, the *Oenomscl* fragment was amplified by PCR using the *Oenomscl* forward and reverse primers. Agarose gel electrophoresis results (Figure S1A) showed a single band of the expected size (lane 3–4, 390 bp). The recombinant plasmid was formed by ligation with the linearized pIB184 vector after restriction digestion and transformed into *E. coli* Top10. Colony PCR identification of positive clones (Figure S1B) showed that lanes 5, 6, 9, and 12 yielded amplification fragments of the expected size. The recombinant plasmid from a positive clone was extracted and sequenced. The correctly sequenced recombinant plasmid was electroporated into *O. oeni* SD-2a cells. *O. oeni* transformants are shown in Figure S1C. Colony PCR identification of positive clones (Figure S1D) showed that lanes 1, 3, 4, and 5 yielded amplification fragments of the expected size, confirming the successfully transformed *Oenomscl* overexpression strain for subsequent experiments. The construction scheme for the *Oenomscl* overexpression strain is shown in Figure 1.

### 3.2. Overexpression of Oenomscl Specifically Enhances Sensitivity of O. oeni Under Specific Acid Stress Conditions

**Figure 2:**
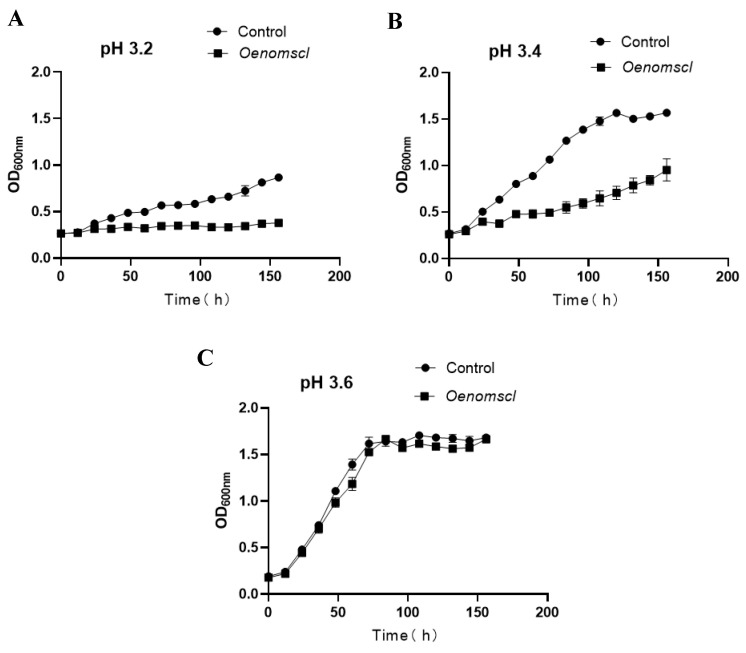
The growth curve of control and *Oenomscl* overexpression strains cultured in FMATB medium at pH 3.2 (**A**), pH 3.4 (**B**), pH 3.6 (**C**) conditions.

To investigate the effect of *Oenomscl* overexpression on the growth of *O. oeni* under acid stress conditions, growth curves of the *Oenomscl* overexpression strain and the control strain were determined under acid stress conditions (pH 3.2, pH 3.4, pH 3.6). The results showed that compared to the control strain, *Oenomscl* overexpression significantly reduced the growth rate of *O. oeni* at pH 3.2 and pH 3.4 (Figure 2A,B) but had no significant effect on the growth rate at pH 3.6 (Figure 2C). This indicates that *Oenomscl* overexpression is not beneficial for *O. oeni* under specific acid stress conditions.

### 3.3. Overexpression of Oenomscl Specifically Enhances the Growth Rate of O. oeni Under 10% Ethanol Stress

**Figure 3:**
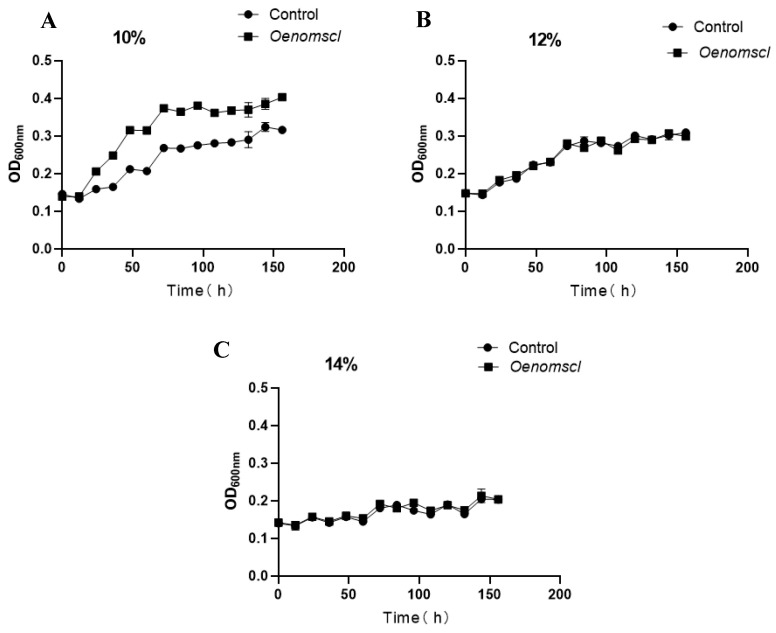
The growth curve of control and *Oenomscl* overexpression strains cultured in FMATB medium at 10% ethanol (*v*/*v*) (**A**), 12% ethanol (*v*/*v*) (**B**), and 14% ethanol (*v*/*v*) (**C**) conditions.

To investigate the effect of *Oenomscl* overexpression on the growth of *O. oeni* under ethanol stress conditions, growth curves of the *Oenomscl* overexpression strain and the control strain were determined under ethanol stress conditions (10%, 12%, 14% *v*/*v*). Overexpression of *Oenomscl* significantly increased the growth rate of *O. oeni* under 10% ethanol stress (Figure 3A). However, when the ethanol stress concentration was further increased (12% or 14%), this promoting effect was no longer significant (Figure 3B,C). Combined analysis of the acid stress experimental results indicates that the effect of *Oenomscl* overexpression on *O. oeni* varies depending on the stress condition, and the expression level of *Oenomscl* needs to be finely tuned, as overexpression does not necessarily help *O. oeni* resist all stress conditions.

### 3.4. Overexpression of Oenomscl Significantly Alters Cell Membrane Properties of O. oeni at Different Growth Stages Under 10% Ethanol Stress

**Figure 4:**
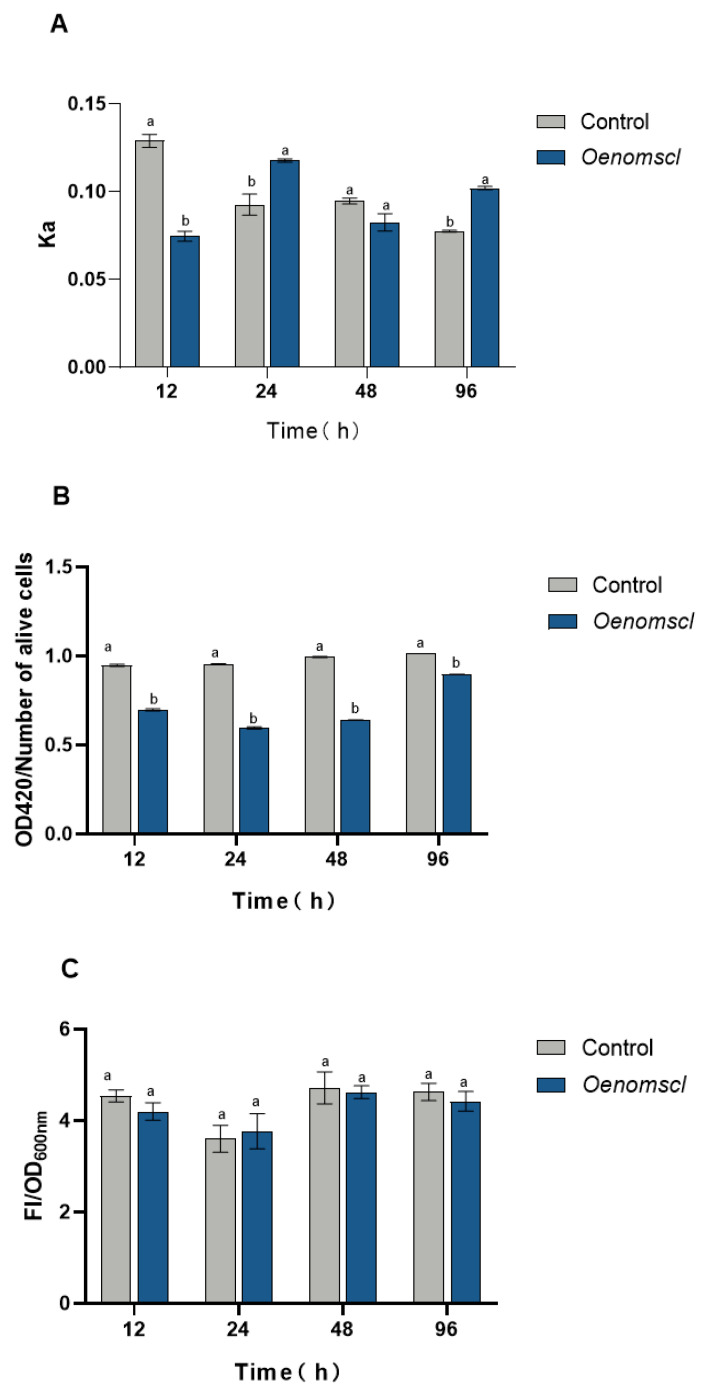
Measurement of cell membrane fluidity (**A**), permeability (**B**), and integrity (**C**) in control and *Oenomscl* overexpression strains cultured under 10% ethanol (*v*/*v*) conditions for 12 h, 24 h, 48 h, and 96 h. Lowercase letters a and b represent a T-test *p*-value < 0.05. Lowercase letters a and a represent a T-test *p*-value ≥ 0.05.

Ethanol stress can significantly alter the physiological state of the *O. oeni* cell membrane. To further investigate the effect of *Oenomscl* overexpression on the cell membrane state of *O. oeni* under 10% ethanol stress, cell membrane fluidity, permeability, and integrity were measured in the overexpression and control strains at 12 h, 24 h, 48 h, and 96 h of growth under 10% ethanol stress. To clarify the transcriptional level of *Oenomscl* in the overexpression and control strains at these different time points, quantitative real-time PCR was performed. The results showed that the expression levels of *Oenomscl* in the overexpression strain at 12 h, 24 h, 48 h, and 96 h were 1.2-, 1.3-, 3.7-, and 0.9-fold those of the control strain, respectively (Figure S2), indicating that, except at 96 h, the expression level of *Oenomscl* was higher in the overexpression strain than in the control strain at the other time points. Cell membrane fluidity measurements showed that at 12 h of ethanol stress, the membrane fluidity of the overexpression strain was significantly lower than that of the control strain, whereas at 24 h and 96 h, the membrane fluidity of the overexpression strain was significantly higher than that of the control strain (Figure 4A). This indicates that *Oenomscl* overexpression significantly altered the cell membrane fluidity of *O. oeni* at different time points, which may influence the adaptation of the overexpression strain to ethanol stress. Cell membrane permeability assays showed that the membrane permeability of the overexpression strain was significantly lower than that of the control strain at all four time points (Figure 4B), indicating that *Oenomscl* overexpression significantly enhanced the control of cell membrane permeability in *O. oeni*. Cell membrane integrity assays showed no significant difference between the overexpression and control strains at the four time points (Figure 4C), suggesting that *Oenomscl* overexpression had no significant effect on cell membrane integrity under 10% ethanol stress. These results indicate that *Oenomscl* overexpression enhances the adaptation to ethanol stress by enhancing the control of cell membrane permeability at different time points and altering cell membrane fluidity at these time points.

### 3.5. Overexpression of Oenomscl Significantly Alters the Protein Expression Pattern of O. oeni Under Ethanol Stress

**Figure 5:**
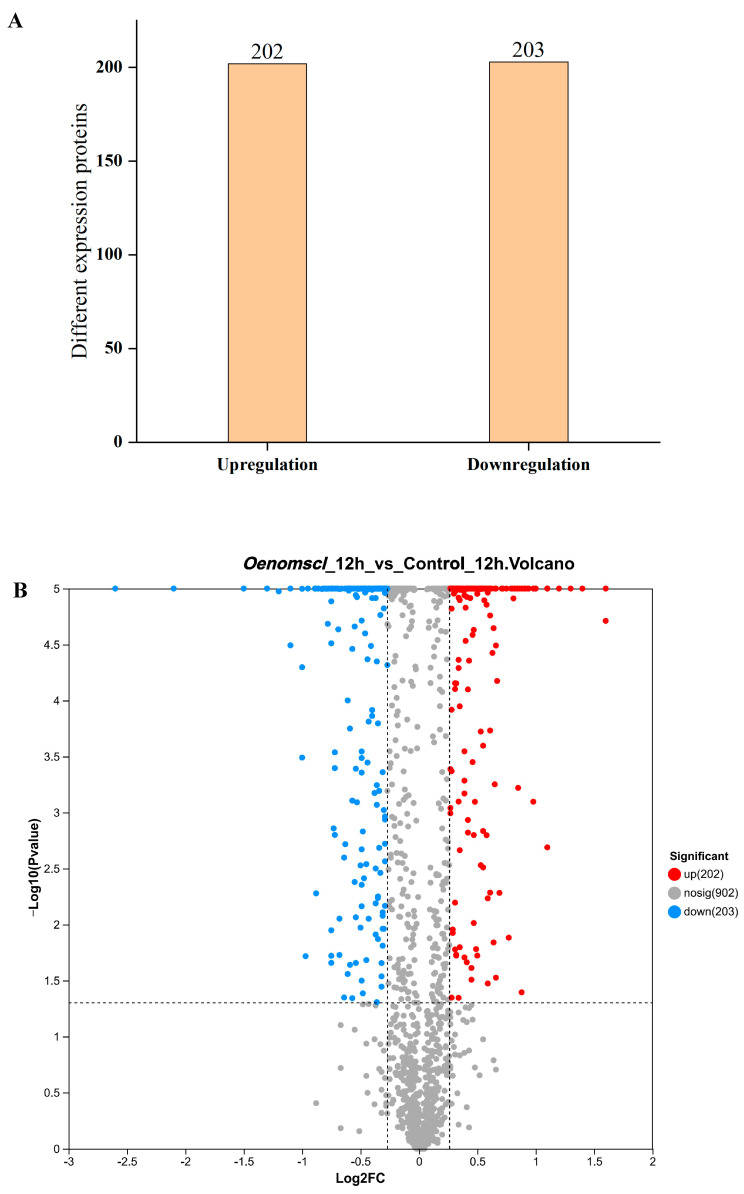
Number of differentially expressed proteins (**A**) and volcano plots of protein expression differences (**B**) between control and *Oenomscl* overexpression strains cultured in FMATB medium with 10% ethanol (*v*/*v*) at 12 h. The dashed lines in B are the boundaries that separate the distribution regions of proteins with no significant differential expression from those with significant differential expression.

**Figure 6:**
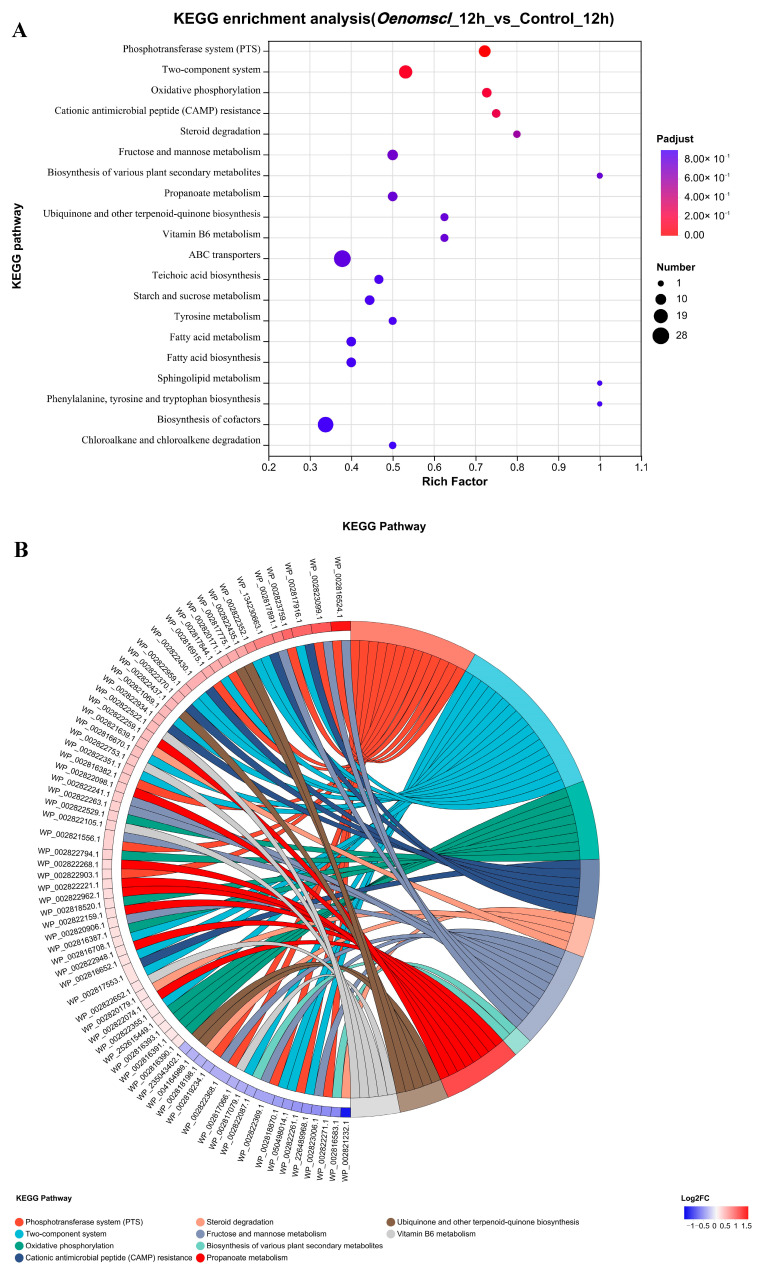
KEGG pathway enrichment bubble chart (**A**) and chord diagram (**B**) for differentially expressed proteins between control and *Oenomscl* overexpression strains. In the bubble chart, the *x*-axis represents the enrichment ratio, and the *y*-axis represents KEGG pathways. Each bubble represents a KEGG pathway. The size of the bubble indicates the number of proteins enriched in that KEGG pathway. Different colors of bubbles represent Padjust values. The chord diagram shows the correspondence between the annotation and enrichment of the differentially expressed protein set and KEGG pathways. Proteins are shown on the left, ordered by log_2_FC from top to bottom; higher log_2_FC indicates greater upregulation, lower log_2_FC indicates greater downregulation, and log_2_FC closer to 0 indicates smaller expression fold change. KEGG pathways enriched with target proteins are shown on the right.

To investigate the effect of *Oenomscl* overexpression on protein expression in *O. oeni* under ethanol stress, proteomic expression was analyzed in samples from the *Oenomscl* overexpression strain and control strain grown under 10% ethanol stress for 12 h. The results showed that compared to the control strain, the *Oenomscl* overexpression strain exhibited 405 differentially expressed proteins at 12 h under 10% ethanol stress, of which 202 were upregulated and 203 were downregulated (Figure 5). Protein function enrichment analysis showed that the differentially expressed proteins were mainly classified into pathways such as ABC transporters, Biosynthesis of cofactors, Two-component system, phosphotransferase system (PTS), and fructose and mannose metabolism (Figure 6). The top 10 most significantly upregulated and downregulated proteins in the *Oenomscl* overexpression strain compared to the control strain are shown in Table S1. *Oenomscl* overexpression primarily affected the expression levels of proteins involved in carbohydrate transport and metabolism, cell wall/membrane/envelope biogenesis, lipid transport and metabolism, amino acid transport and metabolism, and transcription. Notably, membrane transporter proteins constituted a large proportion of the most significantly affected differentially expressed proteins, indicating that *Oenomscl* overexpression significantly impacts the expression levels of proteins related to cell membrane transport and cell wall synthesis.

## 4. Discussion

Directed evolution is a commonly used method to obtain mutants with significant tolerance to stress conditions. Combining genome sequencing and comparative genomics to identify mutation sites in beneficial mutants can provide target genes for targeted genetic manipulation. The *Oenomscl* gene investigated in this study was identified during the analysis of mutation sites in an acid-tolerant mutant. Our research group previously heterologously expressed several *O. oeni* genes potentially involved in stress tolerance in *Lactobacillus plantarum*, a system with more established genetic transformation techniques, and found that these genes could significantly enhance the stress tolerance of the recombinant strains [11,12,13]. However, the genetic transformation system for *O. oeni*, the primary executor of malolactic fermentation in wine, has remained less developed. Several research groups worldwide have attempted to transfer recombinant plasmids into *O. oeni* cells using various genetic recombination techniques and transformation methods, with some successful reports. For example, Assad-García et al. improved the electroporation method for *Oenococcus oeni* ATCC BAA-1163 using ethanol as an immediate membrane fluidizer, increasing electroporation efficiency [20]. Darsonval et al. used a vector to express antisense RNA targeting *hsp18* mRNA, which was electroporated into *Oenococcus oeni* strain ATCC BAA-1163, resulting in recombinant strains with lower survival rates under stress conditions compared to the control strain [21]. He et al. overexpressed *lphsp19.3* from *Lactobacillus plantarum* in *Oenococcus oeni* strain HH1 and found that it significantly enhanced ethanol stress tolerance in *O. oeni* [22]. Overall, reports on genetic transformation in *O. oeni* remain relatively scarce, and to date, there are no reports of successful gene knockout in *O. oeni*. Future efforts should focus on further optimizing genetic manipulation methods for *O. oeni*.

In this study, we constructed the recombinant plasmid pIB184-*Oenomscl* using the pIB184 vector and successfully introduced it into *O. oeni* SD-2a cells via electroporation. By examining the tolerance of the recombinant and control strains under acid and ethanol stress conditions, we found that *Oenomscl* overexpression had differential effects on *O. oeni* adaptation to different stress conditions. OD_600nm_ absorbance was used as an indicator for monitoring the growth status of *Oenococcus oeni* in this study. This method is simple and convenient and has been reported in multiple studies to assess the growth of various lactic acid bacteria strains under different stress conditions [22,23], although it does not accurately reflect the number of viable cells in the culture medium. Overexpression of *Oenomscl* significantly reduced the growth rate of *O. oeni* at pH 3.2 and pH 3.4, suggesting that increased *Oenomscl* content under these conditions increases acid stress sensitivity. Studies have shown that a decrease in pH may protonate positively or negatively charged residues in the C-terminal charge cluster RKKEE of the MscL protein, significantly increasing the activation free energy of the MscL channel [18]. This can lead to the ionization of charged residues or lipid head groups, causing conformational changes in MscL and hydrophobic mismatch between the protein and lipids. This suggests that when acid stress reaches a certain level, MscL may become a target, affecting the cell membrane state of *O. oeni*, which could partially explain why the overexpression strain is more sensitive to certain acid stress conditions. Under 10% ethanol stress, *Oenomscl* overexpression significantly enhanced stress tolerance in *O. oeni*, a phenotype opposite to that observed under acid stress. The mechanisms of damage caused by acid stress and ethanol stress to *O. oeni* differ. Ethanol toxicity is generally attributed to its preferential partitioning into the hydrophobic environment of the lipid bilayer, leading to membrane structural disruption and adversely affecting many membrane-associated processes [24]. Studies have shown that when the membrane is stretched, MscL responds to increased membrane tension by undergoing conformational changes that act as a pressure relief valve, protecting the cell from lysis during significant changes in environmental osmotic pressure [16]. The process of membrane damage during ethanol stress may also be sensed by MscL, which then undergoes corresponding conformational changes to mitigate the damage.

Measurements of various cell membrane physiological indices revealed that under 10% ethanol stress, *Oenomscl* overexpression significantly enhanced the control of cell membrane permeability in *O. oeni*. This may be a crucial reason why the overexpression strain grew faster than the control strain under 10% ethanol stress. Graca da Silveira et al. found that exposing *O. oeni* cells to ethanol leads to increased cytoplasmic membrane permeability, thereby enhancing passive proton influx and concomitant loss of intracellular material, indicating that increased membrane permeability due to ethanol stress is a major manifestation of ethanol-induced damage [24]. Overexpression of *Oenomscl* may enhance the perception of ethanol stress, modulating its conformational changes to control the ethanol-induced increase in membrane permeability and reduce the degree of cellular damage. Overexpression of *Oenomscl* significantly altered cell membrane fluidity at different times under ethanol stress, decreasing fluidity at 12 h and increasing it at 24 and 96 h. Membrane fluidity is typically related to the composition and distribution ratio of lipid molecules in the membrane. Initial acid stress treatment usually decreases membrane fluidity and increases the proportion of cyclic fatty acids in the membrane [25]. Ethanol stress can disrupt the lipid bilayer structure, increasing membrane fluidity and permeability [26]. The alteration of *O. oeni* membrane fluidity at different time points by *Oenomscl* overexpression may influence its adaptation to ethanol stress.

Proteomic analysis was performed to examine protein expression in the *Oenomscl* overexpression and control strains after 12 h of 10% ethanol stress. The results indicated that *Oenomscl* overexpression significantly affected the expression of proteins involved in processes such as sugar, amino acid, and lipid transport/metabolism, as well as cell wall/membrane/envelope biogenesis. This is likely related to MscL’s localization in the cytoplasmic membrane as a large-conductance mechanosensitive channel. The most significantly upregulated proteins in the overexpression strain included three subunits of the PTS mannose/fructose/sorbose transporter system, an AraC family transcriptional regulator, a metalloregulator ArsR/SmtB family transcription factor, an NCS2 family permease (involved in nucleoside transport/metabolism), a D-alanine--poly(phosphoribitol) ligase subunit DltC (involved in lipid transport/metabolism), a D-alanyl-D-alanine carboxypeptidase (involved in cell wall synthesis), and an FtsX-like permease family protein (involved in defense mechanisms). Silveira et al., in their study on the effects of ethanol stress on protein expression in *O. oeni*, found that components of the mannose phosphotransferase system (PTS) were absent in *O. oeni* cells adapting to ethanol stress [27], indicating that ethanol stress indeed affects the transport and subsequent metabolism of certain sugars. In this study, *Oenomscl* overexpression enhanced the protein expression levels of a non-glucose PTS transport system, which may affect the utilization strategy of different sugar types by the overexpression strain under ethanol stress. This hypothesis can be supported to some extent by recent transcriptomic studies on *Salmonella Enteritidis* under ethanol stress, which showed that when *Salmonella Enteritidis* is subjected to ethanol stress, some non-glucose PTS transport systems encoding genes, such as the mannose/fructose/glucitol/sorbitol PTS encoding genes, are significantly upregulated, suggesting that they may play a role in the response of *Salmonella Enteritidis* to ethanol stress [28]. AraC family transcriptional regulators typically act as transcriptional activators, controlling various cellular functions such as carbon metabolism and stress response [29]. Studies have shown that the AraC family transcriptional regulator can activate the expression of genes encoding redox proteins such as Mn superoxide dismutase, NADPH-ferredoxin oxidoreductase, and flavodoxins A and B [30,31]. Metalloregulator ArsR/SmtB family transcription factors usually act as transcriptional repressors, regulating intracellular metal ion concentrations under stress conditions [32]. ArsR/SmtB family transcription factors can sense the concentrations of metal ions such as zinc, cadmium, arsenic, and antimony, and bind to them, inducing conformational changes [33]. They then dissociate from DNA, thereby promoting the expression of genes encoding proteins that export, chelate, or alter the oxidation state of metal ions, thus reducing cytotoxicity [33]. Considering the correlation between the function of these two transcriptional regulators and stress conditions, the upregulation of these two proteins in the overexpression strain may influence its response to ethanol stress. D-alanine--poly(phosphoribitol) ligase subunit DltC and D-alanyl-D-alanine carboxypeptidase are involved in the synthesis of cell wall structural components [34,35]. Their increased expression suggests that the overexpression strain enhances the adjustment of its cell wall structure under ethanol stress. Studies have indicated that the FtsX-like permease family is predicted to be transmembrane proteins that can release lipoproteins from the cytoplasm to the periplasm in an ATP-dependent manner [36]. Changes in the expression level of this protein may affect ethanol stress tolerance in *O. oeni*. The most significantly downregulated proteins in the overexpression strain included a LysM peptidoglycan-binding domain-containing protein and a sugar transferase (involved in cell wall synthesis), a predicted NAD(P)H-binding protein of unknown function, an ABC transporter permease and an aminotransferase class I/II-fold pyridoxal phosphate-dependent enzyme (involved in amino acid transport/metabolism), an SDR family oxidoreductase (involved in lipid transport/metabolism), an ECF transporter S component (involved in cofactor transport/metabolism), a post-translational modification protein SPFH domain-containing protein, a ribosomal structural protein 30S ribosomal protein S15, and a sugar porter family MFS transporter (involved in sugar transport). Studies have shown that LysM peptidoglycan-binding domain-containing proteins have various functions in bacteria, acting as cell wall hydrolases involved in cell wall biosynthesis, modification, and degradation [37]. It is also reported that when *Streptococcus pneumoniae* is treated with arachidonic acid, the gene encoding a LysM-domain containing protein is significantly downregulated [38], whereas the *dltC* gene, which is involved in the teichoic acid biosynthesis pathway, is significantly upregulated in *Limosilactobacillus fermentum* under ethanol stress [39], indicating that bacteria can enhance their adaptability to stress conditions by increasing or decreasing the expression levels of genes involved in regulating cell wall synthesis under such conditions. This implies that the downregulation of LysM peptidoglycan-binding domain-containing protein and the sugar transferase in the overexpression strain could affect bacterial cell wall structure, potentially acting in concert with the upregulated proteins involved in cell wall structural component synthesis. The specific effects of other downregulated proteins on the response of *O. oeni* to ethanol stress require further investigation. Interestingly, studies have shown that SPFH domain-containing proteins play a role in membrane microdomain formation and lipid raft-related processes, such as endocytosis and mechanosensation [40], suggesting a potential link with the function of OenoMscL. These results indicate that *Oenomscl* overexpression significantly alters the protein expression pattern of *O. oeni* under ethanol stress, particularly enhancing its ethanol stress tolerance by affecting the expression levels of proteins involved in cell membrane transport and cell wall synthesis.

It is common to see reports on the use of genetic engineering to modify food fermentation-related microorganisms to enhance their stress resistance and fermentation performance; for example, He et al. found that heterologous expression of a small heat shock protein from *Lactobacillus plantarum* in *Oenococcus oeni* HH1 significantly enhanced the recombinant strain’s tolerance to ethanol stress [22]. Our previous research showed that heterologous expression of thioredoxin *trxA* and a two-component signal transduction response transcriptional regulator in *Lactobacillus plantarum* significantly improved the recombinant strain’s acid stress tolerance [12,13]. The *Oenococcus oeni* strain overexpressing the target gene constructed in this study exhibited enhanced tolerance to 10% ethanol stress. These studies all have potential commercial applications, but the use of genetically modified recombinant microorganisms in food processing is currently subject to widespread restrictions. Therefore, exploring safe and reliable genetic modification methods is of great significance for applying useful laboratory findings to food processing practices. Recently, there have been reports on the application of food-grade expression vectors that do not use antibiotics as selection markers; instead, nisin, which is widely recognized as safe, is used as the selection marker [41,42]. In addition, recent reports have described the use of nontransgenic new genomic techniques to modify key genes or promoter regions, thereby enhancing the relevant performance of the modified strains [43]. These techniques can lay a solid foundation for ensuring that genetically modified or engineered *Oenococcus oeni* strains ultimately comply with the Guidelines for the Safety Assessment of Recombinant DNA Microorganisms for Food Use and can be applied to wine brewing and production.

## 5. Conclusions

In this study, by constructing an *Oenomscl* overexpression strain and a control strain and examining their tolerance under different stress conditions, we found that *Oenomscl* overexpression significantly enhances the tolerance of *O. oeni* under 10% ethanol stress. Cell membrane physiological index measurements indicated that overexpression of this gene enhanced the control of cell membrane permeability in the recombinant strain at different time points under ethanol stress, which may be a key reason for the enhanced ethanol stress tolerance. Proteomic analysis revealed that *mscl* overexpression significantly altered the expression levels of some cell membrane transporters and proteins involved in cell wall synthesis. Changes in the expression levels of these proteins may contribute to the enhanced ethanol stress tolerance in *O. oeni*. The proteins identified in this study that may play a role under ethanol stress will serve as clues for further investigation into their specific functions in *O. oeni* under ethanol stress.

## Data Availability

The original contributions presented in this study are included in the article/Supplementary Materials. Further inquiries can be directed to the corresponding author.

## Supplementary Materials

The following supporting information can be downloaded at: https://www.mdpi.com/article/10.3390/microorganisms14050973/s1. Figure S1: Agarose gel electrophoresis results of amplifying *Oenomscl* fragments through PCR reaction (A), colony PCR detection of transformed *Escherichia coli* Top 10 growth clones showed by agarose gel electrophoresis (B), colony growth of *Oenococcus oeni* after electroporation (C) and colony PCR detection of transformed *Oenococcus oeni* growth clones showed by agarose gel electrophoresis(D). Figure S2: The expression level of *Oenomscl* in the *Oenomscl* overexpressing strain relative to the control strain after growing under 10% ethanol stress (*v*/*v*) for 12 h, 24 h, 48 h, and 96 h. Table S1: The top ten significantly up/down regulated proteins in *Oneimscl* over-expression strain compared with control group cultured under 10% ethanol stress (*v*/*v*) condition for 12 h.
