# Supplementary material for: Overexpression of the Large-Conductance Mechanosensitive Channel Gene in Oenococcus oeni Enhances Its Ethanol Stress Tolerance
Source: Microorganisms. 2026 Apr 26;14(5):973. doi: 10.3390/microorganisms14050973 (PMC13209313; doi:10.3390/microorganisms14050973)
Supplement: Supplementary file 1 [file microorganisms-14-00973-s001.zip › microorganisms-4250873-supplementary.pdf]

Table S1. The top ten significantly up/down regulated proteins in *Oneimscl* over-expression strain compared with control group cultured under10% conditions for 12 h.

Related metabolism	Protein annotation	Gene symbol	Relative expression (log_2fc)
**OneimscL over-expression VS control up regulation**			
Carbohydrate transport and metabolism	PTS mannose/fructose/sorbose transporter subunit IIC	LOD97_01900	1.6
	PTS system mannose/fructose/sorbose family transporter subunit IID	LOD97_05555	1.2
	PTS sugar transporter subunit IIB	LOD97_00895	1.1
Transcription	AraC family transcriptional regulator	LOD97_03265	1.6
	metalloregulator ArsR/SmtB family transcription factor	LOD97_01970	1.1
Nucleotide transport and metabolism	NCS2 family permease	LOD97_03400	1.4
Lipid transport and metabolism	D-alanine--poly(phosphoribitol) ligase subunit DltC	LOD97_01210	1.1
Cell wall/membrane/envelope biogenesis	D-alanyl-D-alanine carboxypeptidase	LOD97_03110	1.1
Defense mechanisms	FtsX-like permease family	LOD97_00805	1.1
Function unknown	Hypothetical protein	LOD97_01370	1.3
**OneimscL over-expression VS control down regulation**			
Cell wall/membrane/envelope biogenesis	LysM peptidoglycan-binding domain-containing protein	LOD97_07840	-2.6
	sugar transferase	LOD97_08935	-1.0
General function prediction only	NAD(P)H-binding protein	LOD97_03240	-2.1
Amino acid transport and metabolism	ABC transporter permease	LOD97_02700	-1.5
	aminotransferase class I/II-fold pyridoxal phosphate-dependent enzyme	LOD97_03315	-1.0
Lipid transport and metabolism	SDR family oxidoreductase	LOD97_00125	-1.3
Coenzyme transport and metabolism	ECF transporter S component	LOD97_09070	-1.2
Posttranslational modification, protein turnover, chaperones	SPFH domain-containing protein	LOD97_04775	-1.1
Translation, ribosomal structure and biogenesis	30S ribosomal protein S15	LOD97_05995	-1.1
Carbohydrate transport and metabolism	sugar porter family MFS transporter	LOD97_01960	-1.0

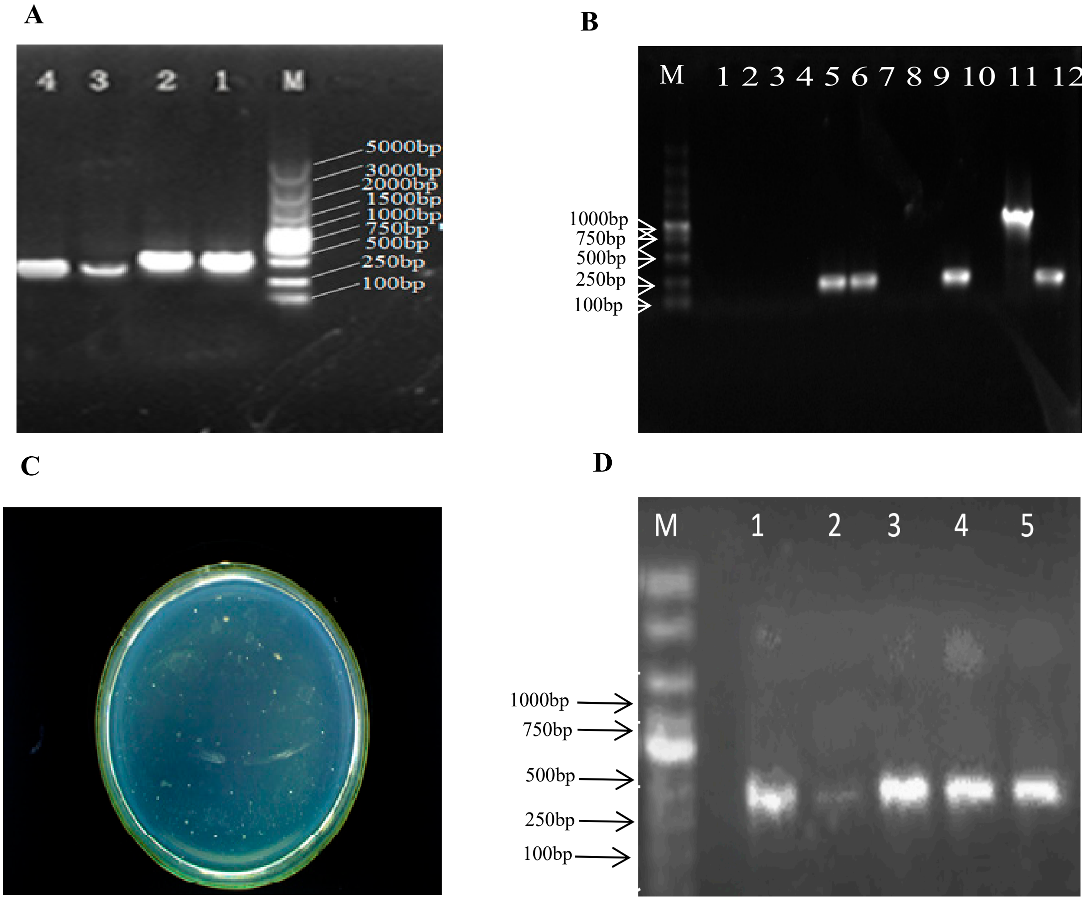

Figure S1. Agarose gel electrophoresis results of amplifying *Oenomscl* fragments through PCR reaction (A), colony PCR detection of transformed *Escherichia coli* Top 10 growth clones showed by agarose gel electrophoresis (B), colony growth of *Oenococcus oeni* after electroporation (C) and colony PCR detection of transformed *Oenococcus oeni* growth clones showed by agarose gel electrophoresis(D). M means marker standard fragments length, the length of some standard fragments are indicated by arrows. The length of *Oenomscl* fragments are 390bp. The Arabic numerals on the top of the different electrophoresis lanes indicate the numbering of the lane order.

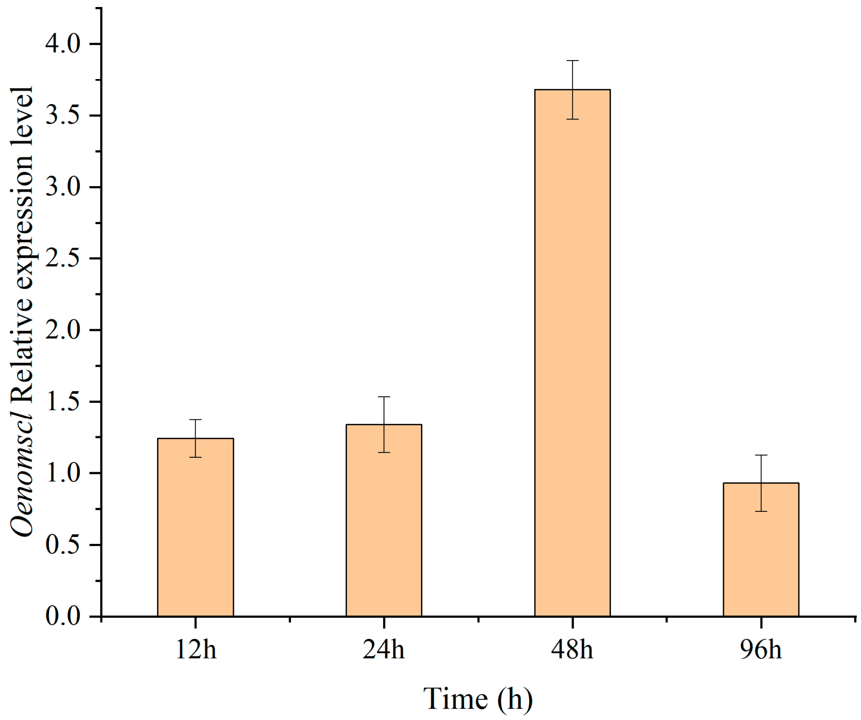

Figure S2. The expression level of *Oenomscl* in the *Oenomscl* overexpressing strain relative to the control strain after growing under 10% ethanol stress for 12h, 24h, 48h, and 96h.
